# Lithium Coupled with C6-Carboxyl Improves the Efficacy of Oligoguluronate in DSS-Induced Ulcerative Colitis in C57BL/6J Mice

**DOI:** 10.3390/md22120573

**Published:** 2024-12-21

**Authors:** Jiayi Li, Meng Shao, Hao Liu, Peng Guo, Fei Liu, Mingfeng Ma, Quancai Li

**Affiliations:** 1 Key Laboratory of Marine Drugs of Ministry of Education, Shandong Key Laboratory of Glycoscience and Glycotechnology, School of Medicine and Pharmacy, Ocean University of China, Qingdao 266003, China; ljy15634420699@163.com (J.L.); mmf621121@163.com (M.M.); 2 Marine Biomedical Research Institute of Qingdao, Qingdao 266071, China; shaomeng@ouc.edu.cn (M.S.); liuhao@ouc.edu.cn (H.L.); guopeng@ouc.edu.cn (P.G.); liufei@ouc.edu.cn (F.L.)

**Keywords:** oligoguluronate, lithium, ulcerative colitis, gut microbiota, collaborative efficacy enhancement

## Abstract

Oligoguluronate lithium (OGLi) was prepared for the purpose of enhancing the anti-ulcerative colitis (UC) activities of OG, in which lithium (Li^+^) is coupled with the C6-carboxyl of G residue. The therapeutic effects of OGLi on dextran sulfate (DSS)-induced UC mice were investigated, and oligoguluronate sodium (OGNa) and lithium carbonate (LC) were used as contrasts. The effects of OGLi, OGNa and LC on the treatment of UC mice were studied by monitoring body weight change and evaluating colon length, the disease activity index (DAI), histopathological examination and gut microbiota regulation. The results showed that compared with OGNa and LC, OGLi significantly reduced the clinical symptoms and histopathological changes associated with UC in the acute model. It was worth noting that OGLi significantly changed the gut microbiota characteristics of the DSS-treated mice and corrected the typical dysbacteriosis of DSS-induced UC. This intervention resulted in increasing the abundance of *norank_f_Muribaculaceae* and *Ileibacterium* spp. while reducing the levels of *Escherichia-Shigella* spp. and *Romboutsia* spp. The OGLi could significantly increase the diversity of intestinal microorganisms in the short term. All of these discoveries demonstrate that lithium collaboratively enhances the anti-UC efficacy of OG, which will help to create OG-based drugs for the treatment of UC.

## 1. Introduction

Ulcerative colitis (UC) is a chronic inflammatory disease characterized by mucosal inflammation that begins in the rectum and often extends to the proximal colon. The primary symptoms of UC include persistent or recurrent diarrhea, abdominal pain and bloody stools [1]. Although the exact cause of UC remains unclear, the interplay of multiple factors, including environmental influences, immune system dysfunction and alterations in the intestinal microbiota, are implicated in the development of the condition [2]. Currently, the primary pharmacological agents employed in the frontline management of UC include 5-aminosalicylic acid (5-ASA), prednisolone and thiopurines. However, these medications exhibit inherent constraints [3]. For example, clinical data indicate that 15% of patients demonstrate intolerance to 5-ASA, and large-scale intestinal microbiota analyses reveal that this intolerance is associated with gut microbial dysbiosis [4]. Numerous studies have indicated that the therapeutic manipulation of gut microbiota via prebiotic intervention is not only a cost-effective and innocuous substitute approach, but also emerges as a promising therapeutic modality for ameliorating UC [5,6].

Alginate is an acidic polysaccharide present in the cell walls of brown algae. It is characterized by an alternating arrangement of β-D-mannuronic acid (M) and α-L-guluronic acid (G) units connected through 1→4 glycosidic bonds [7,8]. In biomedicine, alginate stands out as the most extensively used polysaccharide scaffold in the development of pharmaceutical and medical products, owing to its biological activities and physical properties [9]. For example, alginate can be used as an active pharmaceutical ingredient for the treatment of gastric reflux as well as an excipient for drug sustained release [10,11]. To maximize the pharmaceutical applications of alginate, degradation and chemical modification are two feasible methods to tailor or create new alginate-based bioactive molecules, such as GV-971 (an alginate oligosaccharide for treating Alzheimer’s disease) and PSS (an alginate sulfated derivative as anticoagulant drug) [12], with. Furthermore, alginate is unique in nature, being the only polysaccharide that possesses a carboxyl on each monosaccharide residue. Modulating the salt form coupled with the C6-carboxyl may be another pivotal strategy for enhancing the functional properties of alginate. However, there is little research on this strategy.

The preceding studies examined the activities of oligomannuronate (OM), oligoguluronate (OG) and heterogeneous alginate oligosaccharide (AOS) in mitigating DSS-induced UC in mice by modulating the gut microbiota [13,14,15,16] and found that OG had the potential to be developed as a therapeutic agent for UC. As the currently reported OG existing as sodium salt, we have been interested in exploring whether the salt form coupled with the C6-carboxyl can enhance the anti-UC efficacy of OG. Reportedly, oral lithium formulations have been shown to facilitate colonic regeneration in DSS-induced UC and modulate the gut microbiota composition by enriching short-chain fatty acid-producing bacteria, notably *Akkermansia muciniphila* [17,18]. Building upon these findings, the purpose of this study aims to prepare oligoguluronate lithium (OGLi) and evaluate its enhanced effectiveness in alleviating colitis compared to oligoguluronate sodium (OGNa) and free lithium ion.

## 2. Results and Discussions

### 2.1. Chemical and Characteristic Analysis of OGLi

**Figure 1:**
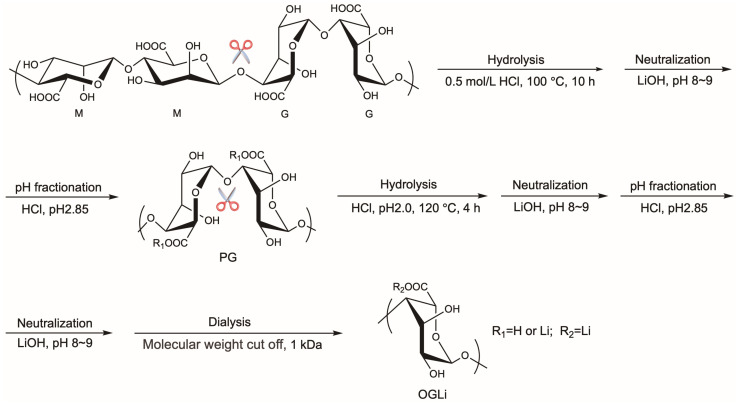
The preparation process of OGLi.

The preparation process of OGLi is shown in Figure 1. The hydrolysis of the interspersed MG-blocks of alginate was the easiest, compared with M-blocks and G-blocks [19]. When alginate was partially hydrolyzed with high acid concentration, MG-block was preferentially disrupted, exposing polymannuronate (PM) and polyguluronate (PG) [19]. M and G were two epimers that differed at C5. The pKa of M was 3.38, whereas that of G was 3.65. At pH 2.85, PM was soluble, whereas PG was insoluble under the same conditions [20]. Therefore, PM and PG could be isolated via pH-fractionated precipitation after the partial hydrolysis of alginate. Because PG had a stiffer chain in water solution, a more severe degradation condition was required for hydrolysis to obtain OG. To obtain high G and a lithium content level of OGLi, a two-stage pH-fractionated precipitation procedure and a three-stage lithium-exchanged procedure were employed.

**Figure 2:**
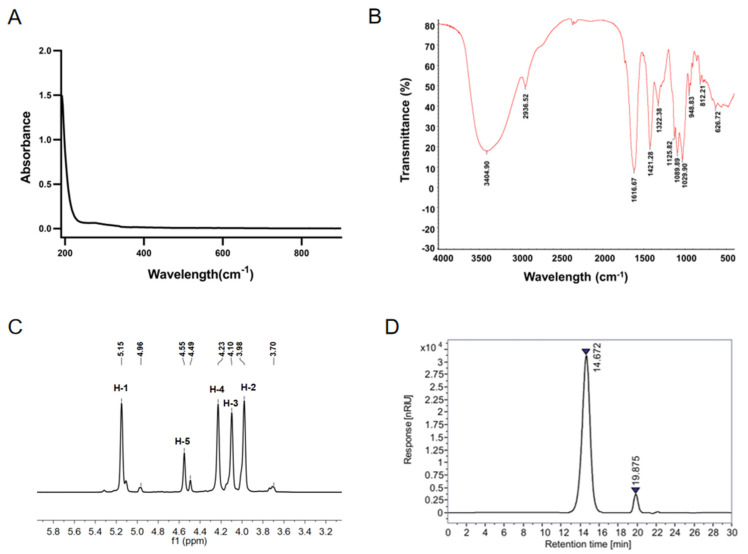
Characteristic analysis of OGLi. (**A**) UV–Vis spectrum. (**B**) FT–IR spectrum. (**C**) ^1^H–NMR spectrum. (**D**) HPGPC chromatogram.

The structure of OGLi was verified based on ultraviolet–visible spectroscopy (UV–Vis), Fourier Transform Infrared spectroscopy (FT–IR) and ^1^H Nuclear Magnetic Resonance spectroscopy (^1^H-NMR). UV–Vis spectrum showed that OGLi revealed end absorption, with the highest absorbance being recorded at 192 nm (Figure 2A). No absorption peak of potential impurities such as protein (~280 nm) and nucleic acid (~260 nm) were observed. The FT-IR spectrum of OGLi (Figure 2B) exhibited prominent absorption peaks at 3404.90 cm^−1^ and 2936.52 cm^−1^, which corresponded to the presence of the hydroxyl group (O-H) and methylene (-C-H) on the oligosaccharides, respectively. The absorption peak at 812.21 cm^−1^ represented to the characteristic δ_C1-H_ of α-guluronic acid residue. The absorption peak at 1616.67 cm^−1^ was the stretching vibration of -C=O. These spectral features unequivocally confirmed the structural composition of OGLi as oligoguluronate [21]. The ^1^H-NMR spectrum of OGLi (Figure 2C) illustrated the chemical shift at 5.15 ppm, attributable to the hydrogen proton located on the anomeric carbon C1 of G residue [22,23]. No obvious chemical shift of M residue was observed, indicating that OGLi had a high G content.

In the HPGPC chromatogram of OGLi (Figure 2D), the peaks at 14.672 min and 19.875 min were attributed to OGLi and NaNO_3_ (the eluent of HPGPC analysis), respectively. No chromatographic peak of other impurities, such as inorganic salts, was observed, indicating that OGLi had a high purity without free Li^+^. The weight-average molecular weight (*Mw*) of OGLi was 3067 Da, calculated with GPC software. The metal element composition of OGLi was determined using Inductively Coupled Plasma Atomic Emission Spectrometer (ICP-AES), and the lithium content was 2.96%. The total concentration of other metal ions (Na^+^, K^+^, Mg^2+^ and Ca^2+^) was 0.23%, indicating that the C6-carboxyl of OGLi existed almost in the form of Li^+^.

### 2.2. Anti-UC Efficacy of OGLi in DSS-Induced UC Mice

To investigate whether Li^+^ at C6-carboxyl improved the efficacy of OG in the DSS-induced UC, OGNa was prepared as the contrast using the same procedure illustrated in Figure 1 except that the LiOH was substituted with NaOH. The *Mw* of the obtained OGNa was 3029 Da, which was in line with OGLi.

**Figure 3:**
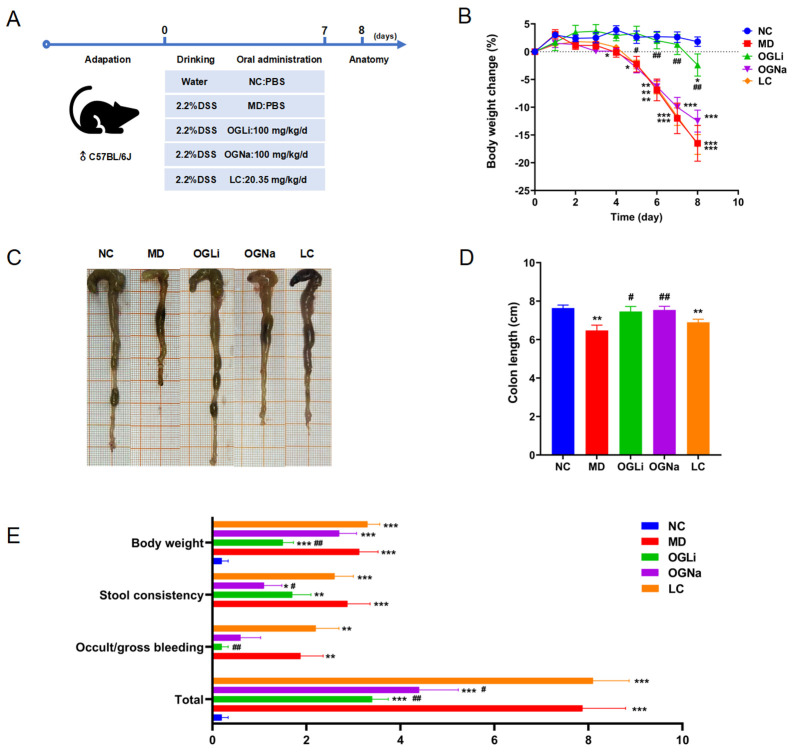
The effects of the oral administration of OGLi, OGNa and LC on DSS-induced UC in mice. (**A**) A graphical illustration of the experimental design. (**B**) Body weight changes of the mice. (**C**) Representative images of the colon. (**D**) The quantitative analysis of colon length. (**E**) The DAI analysis of UC. * *p* < 0.05 versus NC group; ** *p* < 0.01 versus NC group; *** *p* < 0.001 versus NC group; # *p* < 0.05 versus MD group; ## *p* < 0.01 versus MD group.

Based on our previous laboratory investigations [16], we determined that the optimal dose for subsequent animal studies was 100 mg/kg/d of OG, as shown in Figure 3A. Free Li^+^ (Li_2_CO_3_, LC) was used as another contrast. The dose of LC was converted based on the Li^+^ content in 100 mg/kg/d OGLi, with the purpose of comparing the effects of LC and OGLi at the same Li^+^ dose. Except for the control group (NC), 2.2% DSS was added to the drinking water of C57BL/6J mice, resulting in an acute UC model. The protective effects against acute UC were determined by evaluating mouse body weight, colon length and the disease activity index (DAI) and conducting histopathological analyses of colon tissues.

As shown in Figure 3B, a notable reduction in body weight was observed in the model group (MD) compared to the NC group within 4–8 days. It is particularly worth noting that there was no significant difference in body weight between the OGLi and NC groups within 1–7 days and a significant difference between the OGLi and MD groups within 5–8 days, indicating that OGLi could delay the DSS-induced weight loss in mice. Compared to the MD group, there was no significant difference in body weight in OGNa and LC groups within 1–8 days.

At the end of the experiment, colonic bleeding, fecal immobility and reduced colon length were observed in DSS-treated mice of the MD group, as shown in Figure 3C and Figure 3D, respectively. The consumption of OGLi and OGNa could ameliorate this effect. However, there was no significant difference in the symptoms of bleeding, loose stools and shortened colon length between the LC group and the MD group.

The DAI scores revealed that the administration of OGLi significantly reduced UC symptoms such as weight loss, diarrhea and rectal bleeding in mice (Figure 3E). Compared to OGLi, OGNa also significantly reduced UC-induced colon shortening, but it did not demonstrate an improvement in weight loss or rectal bleeding symptoms. However, OGNa had a better efficacy than OGLi in stool consistency. The mice treated with LC at an equivalent lithium concentration of OGLi did not exhibit notable enhancements in DAI scores. In summary, the therapeutic efficacy of OGLi demonstrated superiority over that of OGNa and LC, as evidenced by the composite scores.

**Figure 4:**
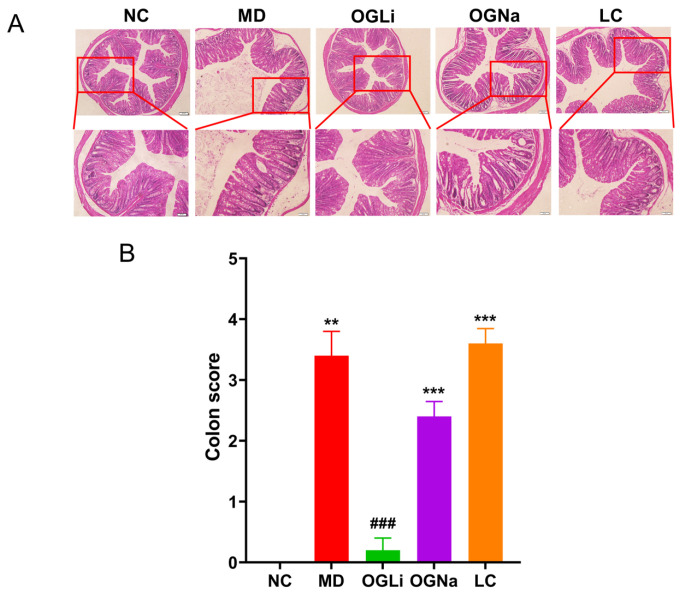
The effects of the oral administration of OGLi, OGNa and LC on DSS–induced mucosal damage in the colon. (**A**) H&E staining. (**B**) Colon score analysis based on H&E staining. ** *p* < 0.01 versus NC group; *** *p* < 0.001 versus NC group; ### *p* < 0.001 versus MD group.

It has been reported that colitis is accompanied by phenotypic and pathological alterations within colonic tissues [3,24]. A hematoxylin and eosin stain (H&E) analysis and a histopathological colitis score analysis of colon specimens revealed that mice induced with DSS manifest pronounced mucosal injury in the distal colon, which is characterized by diminished goblet cells, disrupted epithelial cells and substantial infiltration of inflammatory cells (Figure 4A,B). The mice treated with OGLi and OGNa exhibited relatively preserved colonic tissue architecture and marked amelioration of colonic mucosal damage. Compared to OGNa, OGLi had a more significant efficacy. Nevertheless, the reduced goblet cells, reduced mucosal injury and thickening of the muscular layer after LC treatment were still notably observed.

### 2.3. Gut Microbiota Regulation of OGLi in DSS-Induced UC Mice

**Figure 5:**
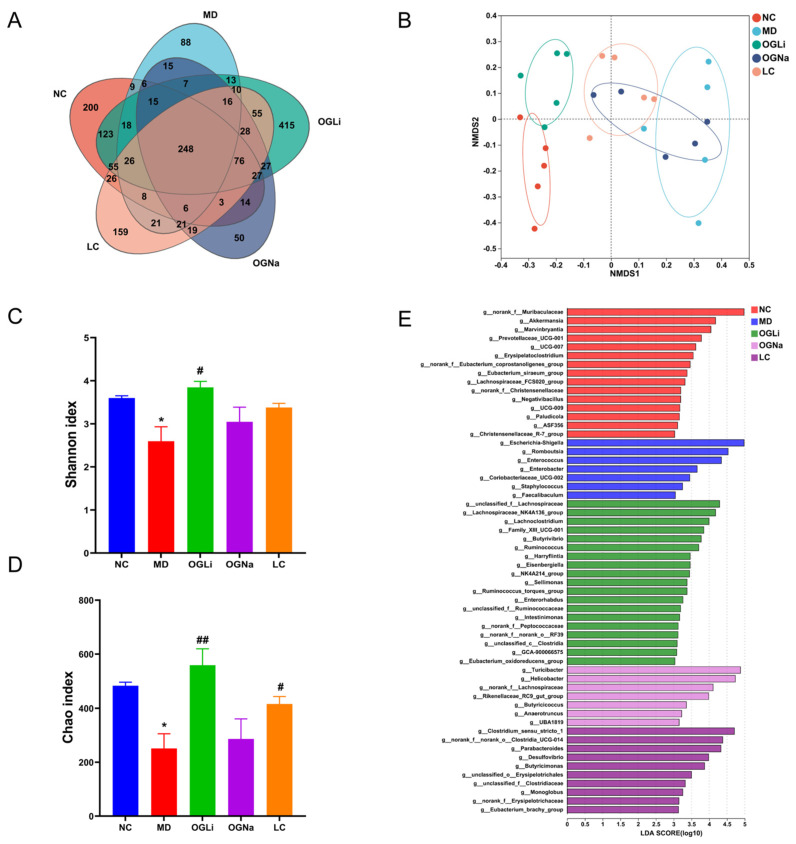
The effects of the oral administration of OGLi, OGNa and LC on the composition of gut microbiota in mice. (**A**) A Venn diagram analysis of the OTUs. (**B**) NMDS score plot analysis. (**C**) The Shannon index of gut microbial diversity and abundance. (**D**) The Chao index of gut microbial diversity and abundance. (**E**) The LEfSe LDA score analysis of the gut microbiota between the five groups. * *p* < 0.05 versus NC group; # *p* < 0.01 versus MD group; ## *p* < 0.001 versus MD group.

In the Venn diagram analysis (Figure 5A), OGLi demonstrated the highest level of intersection compared to the other groups. Non-metric Multidimensional Scaling (NMDS) score plot analyses (Figure 5B) indicated that the oral administration of OGLi, OGNa and LC had a significant impact on the composition of the intestinal microbiota in mice. Meanwhile, the Chao index and Shannon index showed that the alpha diversity of the gut microbiota in mice was the highest in the OGLi group compared to the MD, OGNa and LC groups, even higher than the NC group (Figure 5C,D). In the NC group, the linear discriminant analysis (LDA) levels of *norank_f_Muribaculacea* and *Akkermansia* spp. were in the top two, while that of *Escherichia-Shigella* spp. and *Romboutsia* spp. were in the MD group (Figure 5E). In the OGLi group, the populations of *g_unclassified_f_Lachnospiraceae, Lachnospiraceae_NK4A136_group* spp. and *Lachnoclostridium* spp. were enriched. The abundance of *Turicibacter* spp., *Helicobacter* spp. and *g_norank_f_Lachnospiraceae* increased in the OGNa group. In contrast, LC was associated with an increase in *g_Clostridium_sensu_stricto_1*. These findings indicated substantial disparities in the composition of gut microbiota following the oral administration of OGLi, OGNa and LC.

**Figure 6:**
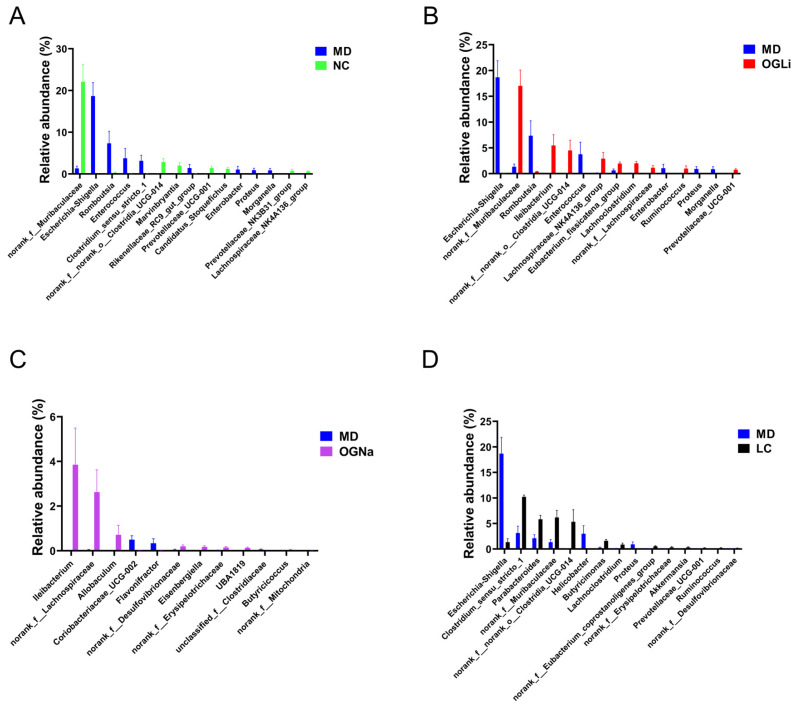
The effects of the oral administration of OGLi, OGNa and LC on statistically significant differences (*p* < 0.05) in the abundance of gut microbiota at the genus level. (**A**) The taxa with significant differences in abundance at the genus level between MD versus NC. (**B**) MD versus OGLi. (**C**) MD versus OGNa. (**D**) MD versus LC.

The key bacterial taxa were identified following OGLi, OGNa and LC treatments at the genus level and compared to the MD group (*p* < 0.05). Significant differences were revealed in the abundance of 15 genera between the NC and MD groups. The abundance of pathogenic bacteria increased in DSS-induced colitis mice (Figure 6A). Treatment with OGLi induced significant changes in the composition of 15 genera, which counteracted the proliferation of detrimental bacteria like *Escherichia-Shigella* spp. and *Romboutsia* spp. while promoting the colonization of *norank_f_Muribaculaceae* and *Ileibacterium* spp. (Figure 6B). Remarkably, comparative analyses among the MD, OGNa, OGLi and LC groups illustrated that OGLi and LC groups reduced the population of *Escherichia-Shigella* spp., while the trend was not observed in the OGNa group (Figure 6C,D). Altogether, our study indicated that the presence of lithium ions could reduce the abundance of *Escherichia-Shigella* spp., whose role in the human gut had been proposed as detrimental [25], and this could help us to understand the therapeutic effect of OGLi on chemical-induced colitis in mice.

In clinical practice, lithium salts are used as psychotropic medications to treat chronic mental ailments, bipolar disorders, neurodegenerative conditions and even brain injuries [26,27,28]. Recent reports indicated that oral LC had the potential to modulate the composition of intestinal microbiota by enhancing the proliferation of short-chain fatty acid-producing bacteria like *Akkermansia muciniphila* [18]. These findings corresponded with the research findings of this study, underscoring the ability of oral lithium to augment the levels of *Akkermansia* spp. in the gut. Alginate and its oligosaccharides had demonstrated efficacy in ameliorating colitis, and there was a clear link between gut microbiota dysbiosis and inflammatory bowel disease [29,30]. Our previous studies confirmed the anti-UC properties of PG, pinpointing *Bacteroides xylanisolvens* spp. and *Lactobacillus murinus* spp. as key players in PG-mediated therapy [15,16]. In line with our previous findings, this study found that an augmentation in *Muribaculaceae* spp. abundance in the presence of OG was observed compared to the MD group. Hence, we posit that the synergistic impact of combining OG with lithium potentiates the anti-colitis efficacy of individual dosing at equipotent levels. This study revealed that OGLi, in particular, confers a more pronounced therapeutic advantage in managing UC as opposed to OGNa. By modulating the composition of the intestinal microbiota, OGLi augments the pharmacological efficacy of oligoguluronate in anti-UC interventions.

It is important to note that there are still several unresolved issues that require further exploration and investigation. It is necessary to elucidate how the anti-inflammatory properties demonstrated by OGLi synergizing with specific target strains. At the microbial strain level, it is also crucial to determine the pivotal signaling intermediates generated through bacterial fermentation under OGLi and how it influences host immune regulatory mechanisms.

## 3. Materials and Methods

### 3.1. Chemicals and Reagents

Alginic acid was provided by Qingdao Gather Great Ocean Algae Industry Group Co., Ltd. (Qingdao, China). Lithium carbonate was obtained from Sinopharm Chemical Reagent Co., Ltd. (Shanghai, China). Dextran sulfate sodium was sourced from MP Biomedicals. Oligoguluronic acid standard with different DP were purchased from Qingdao Hehai Biotechnology Co., Ltd. (Qingdao, China). The 4% paraformaldehyde (PFA) solution was acquired from Beijing Regen Biotechnology Co., Ltd.(Beijing, China). The alcian blue staining solution was purchased from Sigma. The hematoxylin and eosin (H&E) staining solution was obtained from Servicebio (Wuhan, China). Unless otherwise specified, all other chemicals of analytical grade were acquired from Sinopharm Chemical (Shanghai, China), and deionized water was used throughout the work.

### 3.2. Preparation of OGLi and OGNa

Alginic acid was suspended in 0.5 mol/L HCl and heated at 100 °C for 7 h. The resulting solution underwent solid–liquid separation to isolate a precipitate. This precipitate was then dissolved in a LiOH solution, while pH levels were maintained at 8–9. Subsequently, the pH of the redissolved solution was adjusted to 2.85 using HCl, followed by another round of solid–liquid separation to yield a precipitate. The precipitate was suspended in HCl, and the final pH was 2.00. The solution was heated at 120 °C for 4 h. After the reaction, the solution was redissolved in a LiOH solution, and the final pH was maintained at 8–9. The pH of the solution was adjusted to 2.85 by adding HCl. The solid–liquid reaction liquid was separated, and the precipitate was obtained. The precipitate was redissolved with LiOH solution, and the pH of the redissolved solution was maintained at 8–9. Ultrafiltration purification was undertaken using the ultrafiltration membrane with a molecular weight cut-off (MWCO) of 1000 Da. The intercepted liquid was freeze-dried to obtain OGLi. OGNa was prepared by substituting LiOH with NaOH and adhering to the identical procedures.

### 3.3. UV–Vis, FT-IR and ^1^H-NMR Spectroscopy

The UV–Vis spectrum of OGLi was determined on a METASH UV-6000PC spectrophotometer (Shanghai Metash Instruments Co., Ltd., Shanghai, China), using a quartz cuvette, at wavelengths ranging from 190 to 900 nm. The IR spectrum of OGLi was determined using a NICOLRT iS5 FTIR spectrometer (Thermo Fisher Scientific, Waltham, MA, USA) in the range of 4000 and 400 cm^−1^. The ^1^H-NMR spectrum was determined using an Agilent Pro Pulse 500 MHz spectrometer (Agilent Technologies Inc., Santa Clara, CA, USA).

### 3.4. HPGPC Chromatogram

OGLi was dissolved in 0.2 mol/L NaNO_3_ solution to prepare a 5 mg/mL solution. The chromatographic conditions were as follows: TSKgel G3000PW_XL_ chromatographic column (300 mm × 7.8 mm, 7 µm); 0.2 mol/L NaNO_3_ solution as the eluent; flow rate of 0.5 mL/min; column temperature of 35 °C; and injection volume of 10 µL. Each sample was detected using the refractive index detector for 30 min. The molecular weight of OGLi was determined using the GPC software OpenLab CDS 2.3, with different DP of guluronic acid as control samples.

### 3.5. ICP-AES

The metal element composition of OGLi was determined by iCAPRQ ICP-AES (Thermo Fisher Scientific, Waltham, MA, USA). All analyses were conducted in triplicates.

### 3.6. Animals and Treatment

All specific pathogen-free (SPF) animals were sourced from Beijing Vital River Laboratory Animal Technology Co., Ltd. (Beijing, China) (Certificate No. SYXK (LV) 2021 0009). The animal experiments were approved by the Ethical Committee of Marine Biomedical Research Institute of Qingdao (Permission No. E-MBW1906-2023-08). All procedures were conducted following the Guide for the Care and Use of Laboratory Animals (National Academies Press, 8th edition, 2011).

To mitigate estrogenic influences, 50 eight-week-old male C57BL/6J mice were used to evaluate the anti-colitis properties of the tested samples. After a week-long acclimatization phase, the mice were randomly assigned to five groups, each comprising 10 individuals. The groups were named as follows: Normal Control (NC) group, DSS-induced colitis Control (MD) group, OGLi group (100.00 mg/kg/day), OGNa group (100.00 mg/kg/day) and LC group (20.35 mg/kg/day). The NC group was provided unrestricted access to sterile distilled water, while other groups received a specified concentration of the DSS solution throughout the dosing period. The DSS solution was replenished every other day until the completion of the experiment. Following a one-week acclimatization period, dosing commenced on the first day of model induction and continued for seven days. On the eighth day, all mice were euthanized humanely, and their colons and ceca were harvested for further experimental analyses. The body weight and the stool morphology of the mice were monitored daily at a consistent time, and the parameters representing the DAI in each experimental group were recorded using the scoring system described elsewhere [31]. Additionally, hematoxylin and eosin (H&E) staining was performed on the colonic tissues, and H&E staining sections were subjected to histopathological analyses of colon tissues according to a scoring system described elsewhere [32].

### 3.7. High-Throughput Sequencing and Bioinformatic Analyses

Metagenomic DNA from the intestinal microbiota in animal experiments was extracted from cecum samples using the Qiagen QIAamp DNA Stool Mini Kit (Hilden, Germany). The 16S V3-V4 hypervariable genes regions were amplified using universal primers 338F (ACTCCTACGGGAGGAGCAG) and 806R (GGACTACHVGGGTWTCTAAT), as previously described in PCR protocols. Subsequently, the amplicons obtained were sequenced on an Illumina PE300 platform by Majorbio Bio-pharm Biotechnology Co., Ltd. (Shanghai, China). The bioinformatics analysis of the 16S sequencing data, which included Venn diagrams, LEfSe and NMDS score plots, was carried out using the Majorbio Cloud Platform.

### 3.8. Statistical Analyses

The data were presented as mean ± SEM. The Student’s *t*-test and GraphPad Prism 8.0 (San Diego, CA, USA) were utilized for data analysis and graph generation. The results were considered statistically significant at *p* < 0.05: * *p* < 0.05 compared to the NC group; ** *p* < 0.01 compared to the NC group; *** *p* < 0.001 compared to the NC group; # *p* < 0.05 compared to the MD group; ## *p* < 0.01 compared to the MD group; ### *p* < 0.001 compared to the MD group.

## 4. Conclusions

For the purpose of enhancing the anti-UC activities of OG, we explored whether the Li^+^ of C6-carboxyl improved its activities. According to a two-stage pH-fractionated precipitation procedure and three-stage lithium-exchanged procedure, an OGLi with high G content and Li content was prepared. Compared to OGNa and LC, OGLi significantly reduced the clinical symptoms and histopathological changes associated with UC. Notably, OGLi significantly changed the gut microbiota characteristics of the DSS-treated mice and corrected the typical dysbacteriosis of DSS-induced UC. In summary, lithium and OG can synergistically enhance efficacy, and OGLi proposed an innovative approach to developing UC treatments.

## Data Availability

The data are available on request from the corresponding authors.
